# Differential Effect of Deleting Members of African Swine Fever Virus Multigene Families 360 and 505 from the Genotype II Georgia 2007/1 Isolate on Virus Replication, Virulence, and Induction of Protection

**DOI:** 10.1128/jvi.01899-21

**Published:** 2022-03-23

**Authors:** Anusyah Rathakrishnan, Samuel Connell, Vlad Petrovan, Katy Moffat, Lynnette C. Goatley, Tamara Jabbar, Pedro J. Sánchez-Cordón, Ana L. Reis, Linda K. Dixon

**Affiliations:** a The Pirbright Institute, Pirbright, Woking, Surrey, United Kingdom; University of North Carolina at Chapel Hill

**Keywords:** African swine fever virus, MGF360, MGF505, vaccine, type I interferon, multigene family, macrophages, protection, viral replication, virulence factors

## Abstract

African swine fever virus multigene family (MGF) 360 and 505 genes have roles in suppressing the type I interferon response and in virulence in pigs. The role of the individual genes is poorly understood. Different combinations of these genes were deleted from the virulent genotype II Georgia 2007/1 isolate. Deletion of five copies of MGF 360 genes, MGF360-10L, -11L, -12L, -13L, and -14L, and three copies of MGF505-1R, -2R, and -3R reduced virus replication in macrophages and attenuated virus in pigs. However, only 25% of the immunized pigs were protected against challenge. Deletion of MGF360-12L, -13L, and -14L and MGF505-1R in combination with a negative serology marker, K145R (GeorgiaΔK145RΔMGF(A)), reduced virus replication in macrophages and virulence in pigs, since no clinical signs or virus genome in blood were observed following immunization. Four of six pigs were protected after challenge. In contrast, deletion of MGF360-13L and -14L, MGF505-2R and -3R, and K145R (GeorgiaΔK145RΔMGF(B)) did not reduce virus replication in macrophages. Following immunization of pigs, clinical signs were delayed, but all pigs reached the humane endpoint. Deletion of genes MGF360-12L, MGF505-1R, and K145R reduced replication in macrophages and attenuated virulence in pigs since no clinical signs or virus genome in blood were observed following immunization. Thus, the deletion of MGF360-12L and MGF505-1R, in combination with K145R, was sufficient to dramatically attenuate virus infection in pigs. However, only two of six pigs were protected, suggesting that deletion of additional MGF genes is required to induce a protective immune response. Deletion of MGF360-12L, but not MGF505-1R, from the GeorgiaΔK145R virus reduced virus replication in macrophages, indicating that MGF360-12L was most critical for maintaining high levels of virus replication in macrophages.

**IMPORTANCE** African swine fever has a high socioeconomic impact and no vaccines to aid control. The African swine fever virus (ASFV) has many genes that inhibit the host’s interferon response. These include related genes that are grouped into multigene families, including MGF360 and 505. Here, we investigated which MGF360 and 505 genes were most important for viral attenuation and protection against genotype II strains circulating in Europe and Asia. We compared viruses with deletions of MGF genes. Deletion of just two MGF genes in combination with a third gene, K145R, a possible marker for vaccination, is sufficient for virus attenuation in pigs. Deletion of additional MGF360 genes was required to induce higher levels of protection. Furthermore, we showed that the deletion of MGF360-12L, combined with K145R, impairs virus replication in macrophages in culture. Our results have important implications for understanding the roles of the ASFV MGF genes and for vaccine development.

## INTRODUCTION

African swine fever virus (ASFV) causes an acute hemorrhagic fever, African swine fever (ASF), in domestic pigs and wild boar that can result in case fatality approaching 100%. The continuing spread of ASF in Africa, Europe, Asia, and most recently the Dominican Republic has a huge socioeconomic impact and threatens global food security. Following the introduction to China in 2018, a reduction in the Chinese pig herd of up to 50% was reported, representing about a quarter of the global pig population (OIE WAHIS, FAO Empress). Outbreaks also continue in domestic pigs in Europe and Africa and in wild boar in many European countries. No vaccines are available to aid control.

ASFV is a large, double-stranded DNA virus that replicates in the cytoplasm of infected macrophages and is the only member of the *Asfarviridae* family (1). The genome contains 170 to 190 open reading frames (ORFs). These code for proteins that are essential for virus replication and transcription, as well as for virus assembly. Many ORFs code for proteins that are not essential for replication in cells but have important roles in host interactions, including evasion of host defenses. However, many of these proteins are poorly characterized or have unknown functions. ASFV-encoded proteins have been identified that inhibit type I interferon (IFN) responses and other host defense pathways, including apoptosis and stress-induced responses (2).

Among the nonessential ORFs are members of five multigene families (MGFs) (3) that have evolved by gene duplication on the ASFV genome. These can vary in copy numbers between isolates. Most of the MGF360 and MGF505 genes are located close to the left genome terminus, but a few are close to the right genome terminus. Analysis of complete genome sequences available identified 19 paralogs of MGF360 and 10 of MGF505, and these vary in number between isolates. Of particular interest is the finding that some low virulence field isolates of ASFV, including OURT88/3 and NHP68, have a large deletion close to the left genome end (4, 5) that removes ORFs MGF360-10L, -11L, -12L, -13L, and -14L and MGF505-1R and -2R and has interrupted MGF505-3R and MGF360-9L. Targeted gene deletions of multiple copies of MGF360 and MGF505 ORFs also attenuates virulent viruses. These large deletions include all seven of those deleted from OURT88/3 and interruptions of MGF360-9L and MGF505-4R ORFs from the genotype I Benin 97/1 (6). A smaller deletion of six copies of these genes (MGF360-12L, -13L, and -14L and MGF505-1R, -2R, and -3R) from a genotype II virus Georgia 2007 also attenuated the parental virulent virus (7). Deletions of multiple copies of MGF360 and MGF505 genes were shown to correlate with increased expression of type I IFN or interferon stimulated genes (ISGs) in infected macrophages compared to the parental virulent virus (6, 8). This indicates that MGF360 and 505 genes play a role in the attenuation of ASFV in pigs and suppression of the type I IFN response. However, the role of different copies of these genes in these processes has been little studied. Deletion of MGF360-1L or MGF360-16R did not reduce virus replication in macrophages or virus virulence in pigs (9, 10). The MGF360-16R protein was shown to interact with host proteins SERTA Domain 3 (SERTAD3) and syndecan-binding protein (SDCBP) (10). Genotype II ASFV with MGF505-7R deleted, on the other hand, was partially attenuated depending on the dose administered (11, 12). MGF505-7R has been demonstrated to inhibit the cGAS-STING signaling via degradation of STING using the autophagy pathway (11) and more recently has been shown to inhibit interleukin 1β (IL-1β) and type I IFN signaling pathways and the JAK-STAT signaling pathway (12, 13). It was proposed that MGF360-12L inhibits NF-κB-mediated signaling by interacting with and targeting importin-α, effectively limiting nuclear translocation of the transcription factor (14). A528R was also shown to inhibit NF-κB nuclear translocation. MGF505-11R was shown to inhibit the cGAS-STING signaling pathways (15, 16).

Here, we deleted the same five MGF360 genes, three MGF505 genes, and interrupted MGF360-9L and MGF505-4R in the virulent genotype II Georgia 2007/1 isolate (GeorgiaΔMGF) as we previously described in the virulent ASFV genotype I Benin 97/1 isolate (6). Pigs immunized with the GeorgiaΔMGF virus did not show clinical signs or viremia, but only 25% protection was conferred after challenge with the parental virulent isolate. Subsequently, we constructed a series of overlapping deletions of MGF360 and 505 genes from Georgia 2007/1 virus from which K145R gene was also deleted (17) to determine whether some of these MGF360 or MGF505 genes may have a greater impact than others on virus replication and attenuation in pigs. ASFV K145R is an immunogenic protein (18, 19) and of interest since it may provide a negative serology marker to distinguish vaccinated from infected pigs (DIVA) in vaccination campaigns. We recently demonstrated that deletion of K145R had a mildly attenuating effect when deleted together with DP148R gene from Georgia 2007/1 (17). Here, the deletion of K145R and one set of four MGF genes (MGF360-13L and -14L and MGF 505-2R and -3R), in ΔMGF(B) mutant did not further attenuate the Georgia isolate. In contrast, an overlapping deletion of four genes in ΔMGF(A) (MGF360-12L, -13L, and -14L and MGF505-1R) or a subset of two of these MGF genes, MGF360-12L and MGF505-1R, reduced virus replication in macrophages and dramatically attenuated the GeorgiaΔK145R virus. Greater antibody and cellular immune responses and protection were induced by the GeorgiaΔK145RΔMGF(A) deletion compared to the virus with deletion of MGF360-12L, MGF505-1R from GeorgiaΔK145R. Comparison of the replication of these and two viruses with MGF360-12L or MGF505-1R singly deleted from GeorgiaΔK145R showed that deletion of MGF360-12L correlated with a reduced replication in macrophages, suggesting an important role for MGF360-12L in efficient virus replication in macrophages and attenuation of infection in pigs.

## RESULTS

### Recombinant gene-deleted viruses.

**FIG 1:**
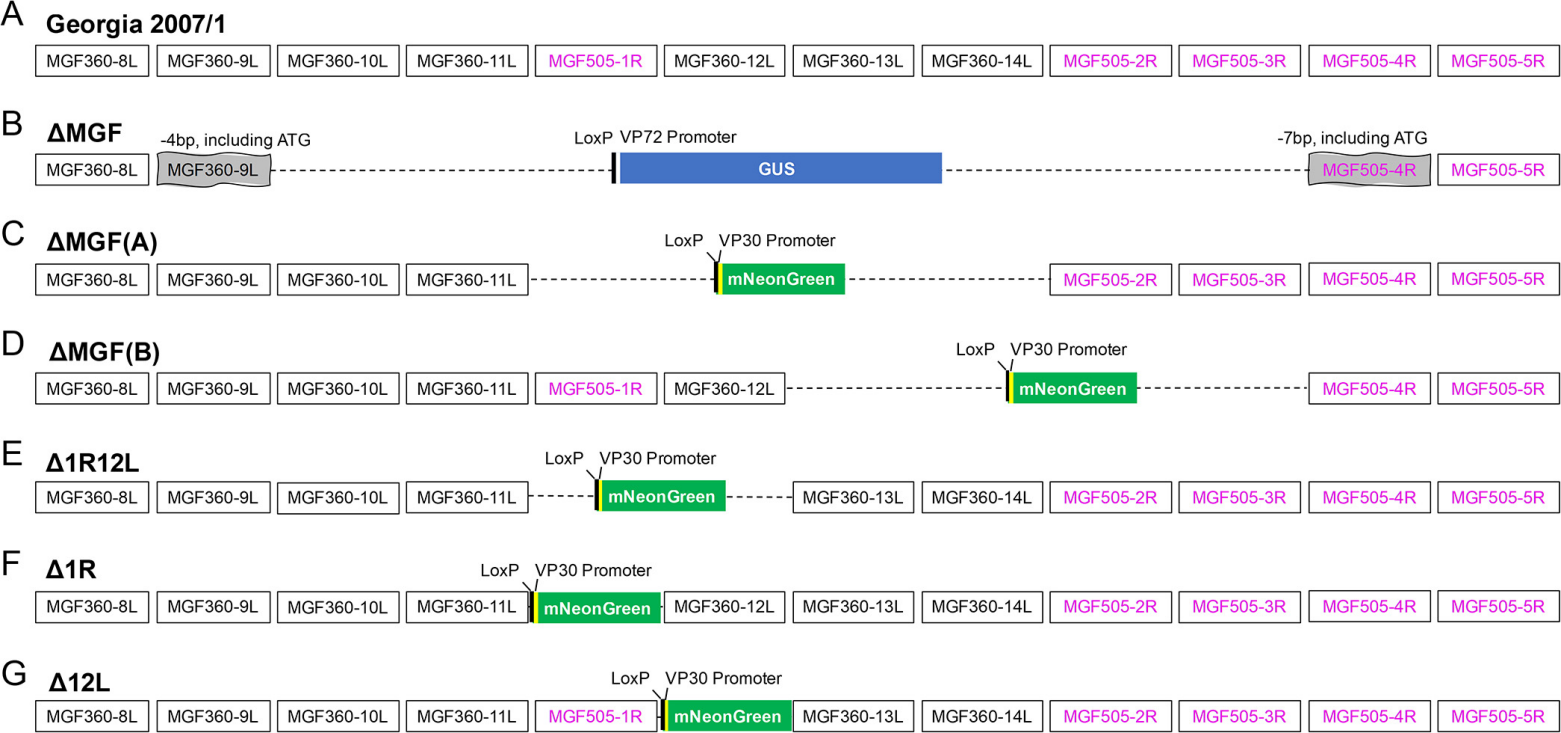
Schematic diagram depicting the deletion of multigene family (MGF) genes. (A) The genome region of African swine fever virus (ASFV) Georgia 2007/1 genotype II strain (accession number FR682468.1) is shown from MGF360-8L to MGF505-5R. The MGF360 genes are outlined in black, and MGF505 genes are outlined in purple. (B) From the same isolate, GeorgiaΔMGF was produced by the interruption of genes MGF360-9L and MGF505-4R (shaded gray) and the deletion of MGF360-10L, -11L, -12L, -13L, -14L, and MGF505-1R, -2R, and -3R. The deleted genes were replaced with the reporter gene GUS under the control of ASFV VP72 promoter (shown in blue). (C) GeorgiaΔK145RΔMGF(A) was produced from GeorgiaΔK145R by deleting MGF505-1R, MGF360-12L, MGF360-13L, and MGF360-14L and replacing these genes with a fluorescent reporter gene, mNeonGreen (shown in green) driven by the ASFV VP30 promoter. To facilitate removal of the reporter cassette by cre-recombinase, loxP sites were added at both sides of the cassette. (D) In the same manner, the recombinant GeorgiaΔK145RΔMGF(B) was produced by the deletion of MGF360-13L, MGF360-14L, MGF505-2R, and MGF505-3R genes and insertion of the mNeonGreen reporter gene under the control of the VP30 promoter. (E) The GeorgiaΔK145RΔMGF360-12LΔMGF505-1R gene-deleted virus was produced by deleting MGF505-1R and MGF360-12L and inserting the mNeonGreen gene under the control of the P30 promoter. (F, G) Single MGF gene deletion viruses GeorgiaΔK145RΔMGF505-1R (F) and GeorgiaΔK145RΔMGF360-12L (G) were produced by deleting either MGF505-1R or MGF360-12L and inserting the mNeonGreen gene under the control of the P30 promoter.

The ΔMGF virus was constructed to delete or interrupt the same genes as those of the genotype I BeninΔMGF virus (6). The BeninΔMGF virus induced moderate clinical signs and viremia postimmunization, with 66 to 100% protection against a homologous virulent isolate depending on the route and dose (20). Our initial expectation was that the same deletion of MGF360 and MGF505 genes from the genotype II Georgia 2007/1 strain would give similar results to the deletion from genotype I Benin 97/1. The deletion of 10,592 bp from Georgia 2007/1 (accession number FR682468.1) (Fig. 1) from positions 25211 to 35802 is shown in Fig. 1B.

However, results from experiment 1 showed that the GeorgiaΔMGF virus was more attenuated and induced lower protection levels compared to the similar BeninΔMGF virus (see below and Table 1). To further investigate which of the MGF360 and MGF505 genes deleted from GeorgiaΔMGF were most important for virus attenuation and induction of protection, we deleted two different combinations of overlapping MGF genes via homologous recombination. To construct the ΔMGF(A) virus, four genes, MGF505-1R, MGF360-12L, MGF360-13L, and MGF360-14L, were deleted from positions 27734 to 32913 (Fig. 1C), whereas for construction of ΔMGF(B), MGF360-13L, MGF360-14L, MGF505-2R, and MGF505-3R were deleted (positions 30595 to 35628) (Fig. 1D). Viruses ΔMGF(A) and ΔMGF(B) contain overlapping deletion of two genes: MGF360-13L and -14L. Both recombinant isolates were produced from a Georgia 2007/1 isolate with the K145R gene deleted. K145R is expressed in the cell cytoplasm late in infection, is an immunogenic protein (18, 19), and was mildly attenuating when deleted in combination with DP148R from Georgia 2007/1 (17). Both viruses were used for immunization of pigs in experiment 2 (see below and Table 1). These viruses were tested for their ability to replicate in macrophages, as well as the level of attenuation in pigs and the induction of protection. The results from experiment 2 showed that ΔMGF(A) was attenuated and induced good levels of protection against challenge, whereas ΔMGF(B) was not significantly attenuated. Therefore, one or both of the two genes uniquely deleted from ΔMGF(A), MGF360-12L, and MGF505-1R are most likely to be responsible for virus attenuation.

**TABLE 1:** Summary of *in vitro* and *in vivo* results using different gene-deleted viruses

Virus	Genes deleted	Growth *in vitro* compared to wild type^*a*^	*In vivo* immunization and challenge			
			Prime^*b*^^,^^*c*^	Boost^*b*^^,^^*c*^	Challenge^*b*^^,^^*c*^	Protection^*d*^
ΔMGF	MGF360: 10L, 11L, 12L, 13L, 14L; MGF505: 1R, 2R and 3R; MGF360-9L and MGF505-4R interrupted	Reduced	i.m 10^4^	i.m 10^4^	i.m 10^4^	25%
ΔMGF(A)	K145R, MGF505-1R, MGF360-12L, MGF360-13L, MGF360-14L	Reduced	i.m 10^4^	First: i.m 10^4^ Second: i.m 10^5^	i.m 10^3^	66.7%
ΔMGF(B)	K145R, MGF360-13L, MGF360-14L, MGF505-2R, MGF505-3R	Not reduced	i.m 10^4^			Culled postimmunization
Δ1R12L	K145R, MGF505-1R, MGF360-12L	Reduced	i.m 10^4^	i.m 10^5^	i.m 10^3^	33.3%
Δ1R	K145R, MGF505-1R	Not reduced	ND^*e*^	ND	ND	
Δ12L	K145R, MGF360-12L	Reduced	ND	ND	ND	

*^a^* The replication level of the recombinant viruses in peripheral blood mononucleocytes is compared to wild-type Georgia 2007/1 virus.

*^b^* i.m indicates an intramuscular route of immunization.

*^c^* The doses are given as 50% haemadsorbing doses (HAD_50_) or 50% tissue culture infective dose (TCID_50_).

*^d^* The percentage of pigs that survived challenge is shown.

*^e^* ND indicates that the value was not determined.

We therefore constructed three other recombinant viruses based on the results of experiment 2. These had deletions of MGF505-1R and MGF360-12L, singly or together from the GeorgiaΔK145R isolate. Δ1R12L has both genes deleted from positions 27734 to 30434 (Fig. 1E) and was used in experiment 3 described below. The final two recombinants either had MGF505-1R (Δ1R) deleted from positions 27734 to 29329 (Fig. 1F) or MGF360-12L (Δ12L) deleted from positions 29382 to 30434 from the GeorgiaΔK145R backbone (Fig. 1G). The growth kinetics of these last two viruses, Δ1R and Δ12L, were compared in peripheral blood mononucleocytes (PBMs) but were not tested by immunization and challenge experiments in pigs. Table 1 summarizes the results obtained.

### Replication of recombinant gene-deleted ASFV in primary porcine bone marrow cells.

**FIG 2:**
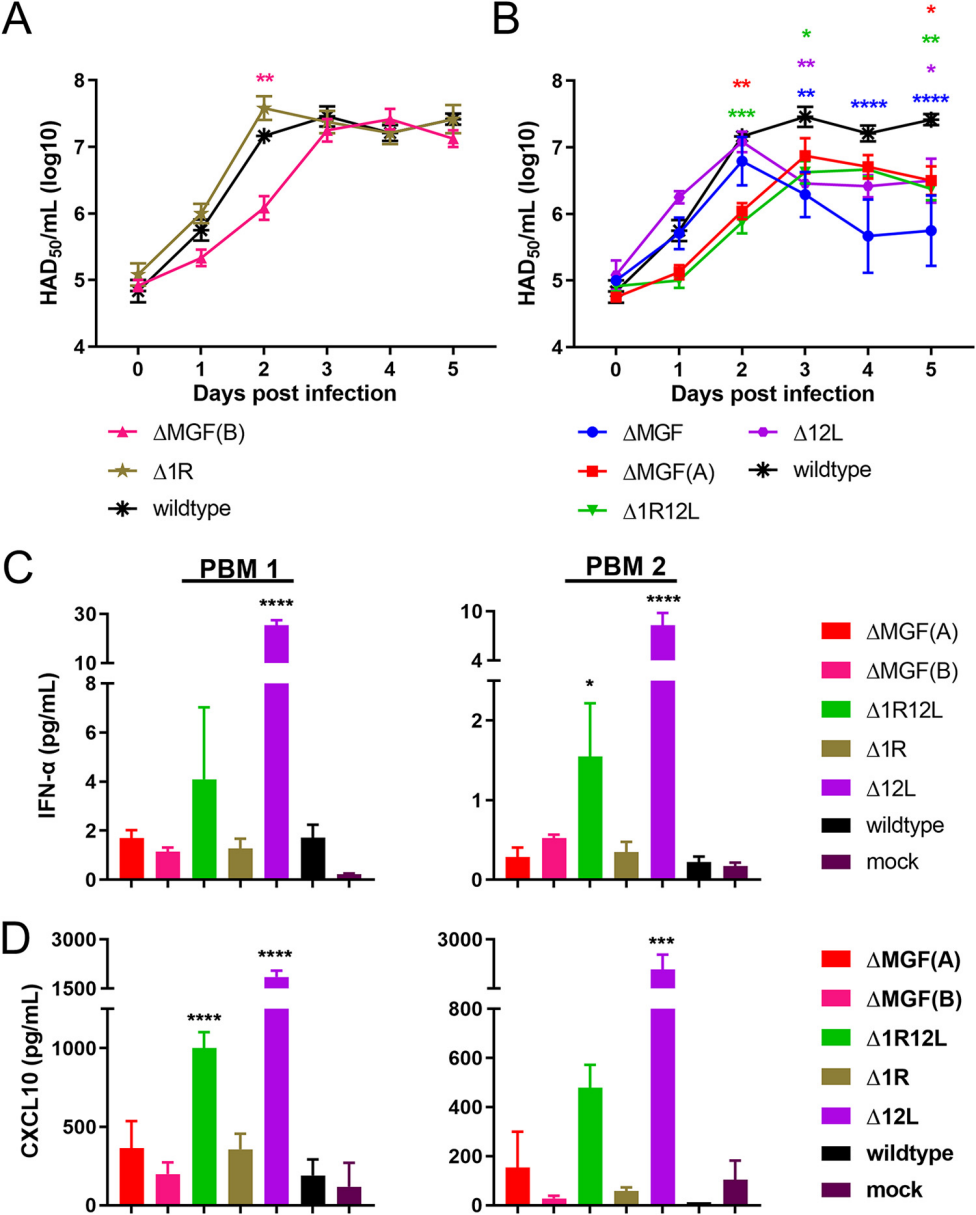
*In vitro* characteristics of recombinant ASFV versus wild-type Georgia 2007/1 isolate. (A, B) Purified peripheral blood mononucleocytes (PBMs) from two different pigs were infected with viruses at multiplicity of infection (MOI) 0.01 in triplicate. Viruses were harvested from both cells and supernatants at different time points and titrated on PBMs in quadruplicate. Multistep growth curves of the gene-deleted viruses are shown over the course of 5 days, where day 0 represents the inoculum. ASFV deletion mutants with similar replication kinetics to Georgia 2007/1 wild type are shown in panel A, while the recombinant viruses with defective replication kinetics are shown in panel B. Two-way analysis of variance (ANOVA) with Dunnett’s multiple-comparison test was performed to evaluate the differences between the recombinant viruses and the wild-type virus. Significant differences are represented by asterisks, where * is *P* < 0.05, ** is *P* < 0.01, *** is *P* < 0.001, and **** is *P* < 0.0001; the color of the asterisk represents the respective virus. (C, D) Purified PBMs from two outbred pigs were infected with viruses or mock at MOI 0.5 in duplicate. Supernatant was collected at 16 h, and the levels of interferon α (IFN-α) (C) and CXCL10 (D) were measured via enzyme-linked immunosorbent assays (ELISAs). The levels of IFN-α (C) and CXCL10 (D) are shown as picogram/mL. One-way ANOVA with Dunnett’s multiple-comparison test was used to compare the levels of IFN-α and CXCL10 expressed in recombinant virus-infected supernatant compared to the wild type-infected supernatant.

The replication kinetics of the six gene-deleted recombinant ASFV in porcine macrophages were compared to virulent Georgia 2007/1 isolate (Fig. 2). PBMs from two different pigs were infected at low multiplicity of infection (MOI) (0.01), and virus from both cells and supernatant combined were harvested every day postinfection for 5 days. The inoculum (represented by day 0 postinfection) showed minimal difference in the starting culture between isolates (Fig. 2). At 24 h postinfection, all seven viruses had similar growth kinetics ranging from 10^5^ to 10^6^ 50% haemadsorbing doses per ml (HAD_50_/mL). Levels of ΔMGF virus peaked at 48 h postinfection at 10^6.8^ HAD_50_/mL and then steadily declined over the next 3 days with significant differences (*P* < 0.001) compared to wild-type Georgia 2007/1 virus (Fig. 2B). This virus had the lowest titer among the six recombinant viruses. The titers of ΔMGF(B) (*P* = 0.0036) (Fig. 2A), ΔMGF(A) (*P* = 0.0023), and Δ1R12L (*P* = 0.0003) (Fig. 2B) were significantly lower than the Georgia 2007/1 wild-type virus titers at 48 h postinfection. However, from day 3 postinfection, ΔMGF(B) had similar titers to the virulent Georgia isolate at ∼10^7^ HAD_50_/mL (Fig. 2A). In contrast, the other two recombinant viruses, ΔMGF(A) and Δ1R12L, maintained slower growth kinetics compared to the wild-type Georgia virus until the end of experiment (Fig. 2B). The single MGF gene deletion virus Δ1R had similar growth kinetics to the wild-type virus (Fig. 2A), whereas Δ12L initially replicated to titers similar to wild-type virus, but from 3 days postinfection, reduced growth was noted and maintained up to day 5 postinfection (Fig. 2B). ΔMGF(A), Δ1R12L, and Δ12L viruses had similar titers to each other and were generally 0.5 to 1.0 log lower that the wild type at 3 to 5 days postinfection.

The results indicate a differential impact of deleting different MGF360 and MGF505 genes on virus growth rates. The viruses that show a reduced growth rate (ΔMGF, ΔMGF(A), Δ1R12L, and Δ12L) all harbor deletion of MGF360-12L gene, suggesting that deletion of this gene plays an important role in the reduced growth rate, although other genes deleted from ΔMGF are likely to contribute.

### Levels of IFN-α and CXCL10 in supernatants from PBMs infected in culture with wild-type and gene-deleted viruses.

Bone marrow cells (PBMs) were infected with wild-type or gene-deleted Georgia 2007/1 viruses at an MOI 0.5 for 16 hours postinfection (hpi). The levels of IFN-α and the interferon-stimulated chemokine CXCL10 in supernatants from infected cells from two different pigs were measured by enzyme-linked immunosorbent assays (ELISAs). The levels detected varied between PBM cells from the two pigs (Fig. 2, PBM 1 or PBM 2). However, consistently the levels of IFN-α in supernatants from cells infected with viruses ΔMGF(A), ΔMGF(B), Δ1R, and Georgia 2007/1 wild type were just slightly higher than in mock-infected cells (Fig. 2C). IFN-α levels were elevated in Δ1R12L-infected cells and greatly increased in cells infected with the single gene deleted, Δ12L at 16 hpi (*P* = 0.0001). Likewise, CXCL10 levels in supernatants from PBMs infected with ΔMGF(A), ΔMGF(B), Δ1R, and Georgia 2007/1 wild type were similar to those in mock-infected samples, whereas levels in Δ1R12L- and Δ12L-infected supernatant were greatly elevated (*P* < 0.001 or *P* < 0.0001) (Fig. 2D).

### Immunization of pigs with recombinant gene-deleted ASFV, followed by challenge with virulent wild-type Georgia 2007/1: clinical observations.

#### (i) Experiment 1.

**FIG 3:**
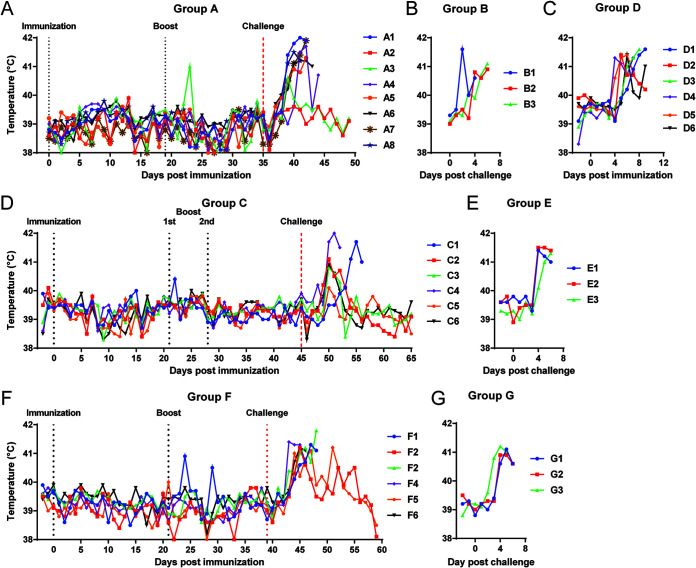
Temperatures of pigs from experiments 1, 2, and 3. (A, C, D, F) Rectal temperatures were recorded daily for pigs immunized with ΔMGF (group A, panel A), ΔMGF(B) (group D, panel C), ΔMGF(A) (group C, panel D), and Δ1R12L (group F, panel F). (B, E, G) The temperatures for the nonimmune challenge control groups for each experiment were recorded following challenge with Georgia 2007/1 isolate.

**FIG 4:**
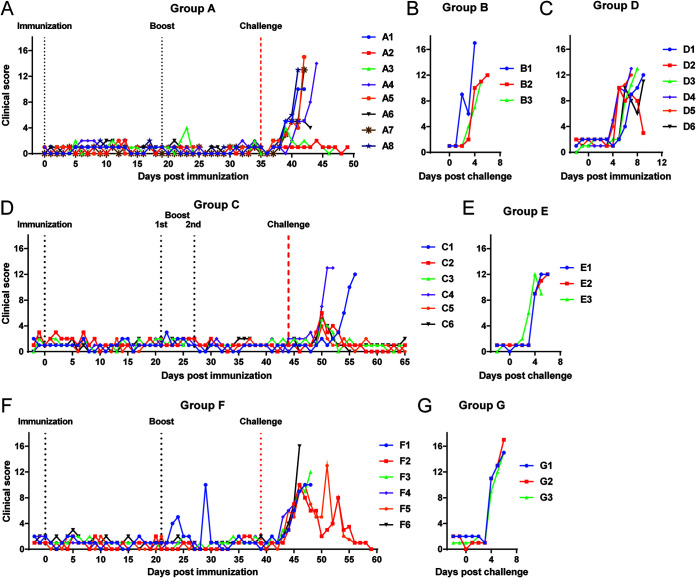
Clinical scores of pigs from experiments 1, 2, and 3. Cumulative clinical scores based on clinical signs observed daily for pigs immunized with (A) ΔMGF (group A), (C) ΔMGF(B) (group D), (D) ΔMGF(A) (group C) and (F) Δ1R12L (group F). (B, E, G) The cumulative scores for the nonimmune, challenge control groups for each experiment were recorded following challenge with Georgia 2007/1 isolate.

A group of eight pigs were immunized i.m with 10^4^ HAD_50_ of ASFV ΔMGF and were boosted with the same virus, at the same dose at day 19 days postimmunization (dpi) (group A). None of the pigs showed clinical signs following immunization or boost (Fig. 3A and 4A) except for one pig that had a temperature of 41°C on 23 dpi but no other clinical signs (Fig. 3A).

All eight pigs, including three nonimmunized pigs (group B), were challenged i.m at 35 dpi with 10^4^ HAD_50_ virulent ASFV Georgia 2007/1. In group A, two pigs survived until the end of the experiment at 14 days postchallenge (dpc). The other pigs developed a temperature above 40.5°C, were lethargic, and refused food starting between 4 and 6 dpc. These pigs reached the moderate severity endpoint between 6 and 9 dpc (Fig. 3A and 4A). The three nonimmune control pigs (group B) had increased temperatures, were lethargic and anorexic between 2 and 5 dpc, and were euthanized at 4 or 6 dpc (Fig. 3B and 4B).

#### (ii) Experiment 2.

To ascertain which of the MGF360 or 505 genes may have contributed to the level of virus attenuation and induction of protections seen in experiment 1, we made two viruses with overlapping deletions of four of the MGF genes, such that two of the genes deleted were in common and two unique for each virus. Two groups of six pigs were immunized with either 1 mL of 10^4^ HAD_50_ ΔMGF(A) (group C) or ΔMGF(B) (group D).

Pigs from group D immunized with a single dose ASFV ΔMGF(B), which has genes K145R, MGF360-13L, MGF360-14L, MGF505-2R, and MGF360-3R deleted, developed signs of acute ASF including increased temperature, lethargy, and loss of appetite (Fig. 3C and 4C). All were euthanized between 7 and 9 dpi. Five of the pigs reached the moderate severity endpoint, while pig D2 without clinical signs or increased temperature was euthanized as it was the only pig left in the group. The clinical signs were slightly delayed compared to control pigs infected with 10^4^ HAD_50_ virulent Georgia 2007/1 virus (group B), which could be attributed to the deletion of K145R, which we previously demonstrated to delay the onset of ASF acute clinical signs (17).

**FIG 5:**
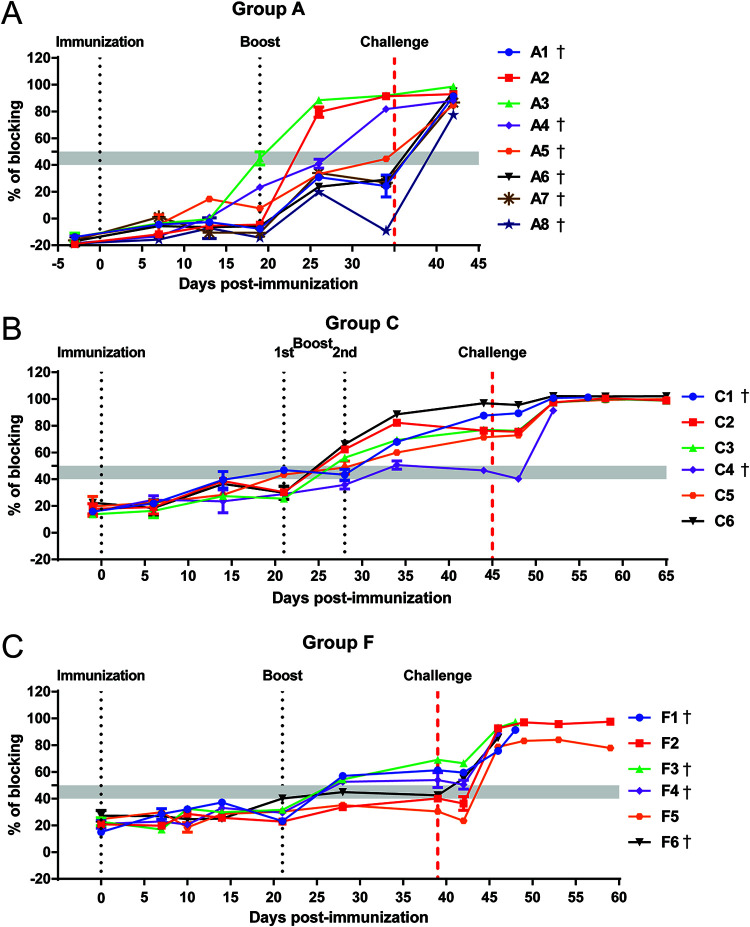
ASFV-specific antibody responses following immunization of pigs. Antibody responses of pigs immunized with ΔMGF (A), ΔMGF(A) (B), and Δ1R12L (C) were measured on different days after immunization and challenge using a blocking ELISA, against ASFV VP72 protein. The results are presented as percentage of blocking, where values above 50% blocking were considered positive antibody responses, while anything below 40% was considered negative. Samples with blocking between 40 and 50% were considered doubtful.

**FIG 6:**
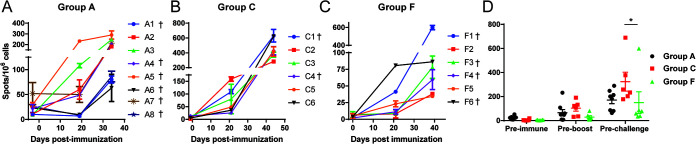
Cellular immune response measured by IFN-γ ELIspot assay following immunization of pigs. The numbers of IFN-γ-producing cells in PBMCs were measured in pigs before or after immunization and boost by ELISpot assays. PBMCs were stimulated with Georgia 2007/1 isolate. The results are presented as mean frequencies of IFN-γ-producing cells per million PBMC of individual pigs. (A to C) The pigs were immunized with either ΔMGF (A), ΔMGF(A) (B), or Δ1R12L (C). (D) Comparison between the three groups.

In group C, the pigs were boosted with the same dose 21 days after the first immunization with ASFV ΔMGF(A), which has genes K145R, MGF505-1R, MGF360-12L, MGF360-13L, and MGF360-14L deleted. None of the pigs displayed any clinical signs postimmunization or postboost. Based on the low antibody and cellular responses observed just before the first boost (Fig. 5B and 6B), an additional boost with the same virus at a higher dose (10^5^ HAD_50_) was given to all six pigs on day 28 pi. None of the pigs showed any clinical signs after the second boost.

On 45 dpi, ΔMGF(A)-immunized pigs and the three nonimmunized control pigs (group E) were challenged with 10^3^ HAD_50_ virulent Georgia 2007/1 virus. Two pigs from group C, C4 and C1, were euthanized at the moderate severity humane endpoint on 7 and 11 dpc, respectively. Pig C1 had delayed clinical signs and a temperature above 40.5°C from 9 dpc compared to 5 dpc in pig C4 (Fig. 3D and 4D). The remaining four pigs survived until termination of the experiment, 20 dpc; therefore, this group had a survival rate of 66.7% after challenge. Three of the survivors, pigs C2, C3, and C6, showed an increase in temperature above 40.5°C at 5 dpc (Fig. 3C). This increased temperature persisted for 2 or 3 days, after which no further clinical signs were observed (Fig. 3D and 4D). Pig C5 did not develop any clinical signs after challenge. The nonimmune pigs in group E all developed high temperatures (40.6 to 41.2°C) at 4 dpc, showed anorexia and were lethargic (Fig. 3E and 4E). All 3 pigs were euthanized at the moderate severity humane endpoint by 6 dpc.

#### (iii) Experiment 3.

Based on Experiment 2, taking into consideration that two of the genes deleted were unique to ΔMGF(A) and two were shared with ΔMGF(B), we attributed the attenuation and protection induced in pigs immunized with ΔMGF(A) to the 2 unique genes deleted from ΔMGF(A), – MGF505-1R and MGF360-12L. We therefore constructed an ASFV Georgia strain lacking K145R, MGF505-1R and MGF360-12L (Δ1R12L). The aim was to confirm if deletion of just these two MGF genes and K145R gene was sufficient to attenuate the virus and induce a protective immune response.

In this experiment, one group of 6 pigs (group F) was immunized with 10^4^ HAD_50_ Δ1R12L. A boost with the same virus at a higher dose (10^5^ HAD_50_) was administered at 21 days after first immunization. None of the pigs developed clinical signs postimmunization. However, 1 pig, F1, had a transient increase in temperature after boost, on days 24 and 29 pi (Fig. 3F). This pig also stopped eating in the afternoon of day 29 (Fig. 4F). Nevertheless, it recovered and remained healthy until challenge.

On 39 dpi, group F and nonimmune pigs (group G) were challenged with 10^3^ HAD_50_ of virulent Georgia 2007/1 virus. All the nonimmune pigs were terminated at day 6 pc after developing typical signs of acute ASFV, including increase in temperature (41.0 to 41.5°C), anorexia, and lethargy (Fig. 3G and 4G). After challenge, only two of the six group F pigs (F2 and F5) survived until the end of the experiment, although both had continuing clinical signs, including sporadic fever and reduced eating until 55 or 52 dpi (15 or 12 dpc) (Fig. 3F and 4F). In addition, pig F5 displayed joint swelling between 11 and 13 dpc. The immunized nonprotected pigs all developed an increase in temperature, refused food, and were lethargic. They were euthanized on 7 and 9 dpc.

### Postmortem pathological observations.

**FIG 7:**
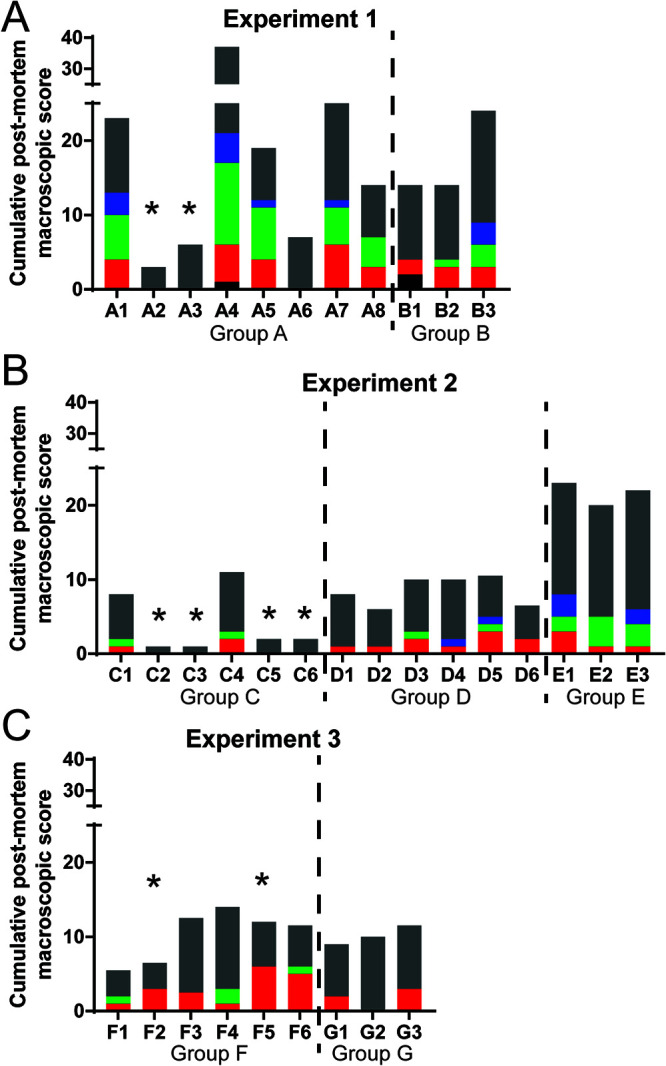
Postmortem macroscopic lesion scoring. Lesions observed were scored and displayed as a cumulative score observed in different organs of the pig (different colored bars). External examination includes general body condition and lesions in eyes, conjunctiva, nostrils, oral cavity, skin, subcutis, skeletal muscles, and joints (black bar). Lesions in the thoracic cavity (red bar) include the presence of thoracic exudates, as well as lesions affecting cardiorespiratory system. Lesions in the abdominal cavity (green bar) include the presence of ascites along with the presence of lesions affecting the gastrointestinal system, including the stomach, intestines, liver, and gallbladder. Lesions observed in the urinary system (kidneys and urinary bladder) are included in the blue bar. Finally, lesions depicted by the gray bar include pathology observed in lymphoid tissues: tonsils, thymus, spleen, and various lymph nodes. The graphs are divided by the experiments whereby panel A is experiment 1, panel B is experiment 2, and panel C is experiment 3. The asterisks shown above the individual bars are results from pigs that were protected against the challenge virus and were terminated at the end of the experiments.

All the pigs immunized with ΔMGF (group A), which did not survive challenge, showed extensive macroscopic lesions typical of acute ASF (Fig. 7A), including hemorrhagic lymph nodes resembling blood clots, enlarged spleen, ascites, and hydropericardium with reddish fluid (total scores, 14 to 37), except for A6, which had a score of 6. The two surviving pigs, A2 and A3, showed enlarged lymph nodes with mild to moderate hemorrhages (Fig. 7A).

Necropsies of pigs immunized with ΔMGF(B) (group D), which were euthanized on 7 to 9 dpi, showed lesions typical of acute ASF (cumulative scores, 6.0 to 10.5), including erythematous tonsils, hydropericardium, enlarged spleen, and lymph nodes increased in size, edematous, and hemorrhagic. Other lesions observed include enlarged heart, mild failure of the lungs to collapse, and petechiae in their kidneys (Fig. 7B). The two group C pigs, immunized with ΔMGF(A) (C4, C1) that were terminated at their moderate endpoints after challenge at 7 and 11 dpc, respectively, had cumulative postmortem lesion scores similar to group D pigs (between 8 and 11) (Fig. 7B). The surviving pigs of group C showed very few lesions (cumulative score, 1 to 2), and they involved slight enlargements of spleen or lymph nodes (Fig. 7B). The nonimmune control pigs in group E, infected with virulent genotype II ASFV, had cumulative scores ranging from 20 to 23. Aside from the characteristic ASF lesions mentioned, the pigs in group E also displayed congestion and edema in lungs, as well as petechiae in the ileum, duodenum, jejunum, kidneys, and/or bladder (Fig. 7B).

The pigs in group F, immunized with Δ1R12L, had a cumulative score ranging from 5.5 to 14 (Fig. 7C). All pigs, including those that survived to the end of the experiment, showed hyperemic splenomegaly. The pigs terminated at the moderate endpoints displayed erythematous tonsils, moderate hydropericardium, ascites, and the presence of some enlarged and hemorrhagic lymph nodes. The lungs of pigs F3 and F6 showed failure of the lungs to collapse, while F6 also had variable degree of congestion and hyperemia. The two surviving pigs, F2 and F5, both displayed areas of pulmonary consolidation. High levels of ASFV p30 antigen were detected by immunohistochemistry (IHC) in pulmonary tissue from pig F2 (data not shown). Histopathologic evaluation of the lungs also revealed the presence of acute nonsuppurative broncho interstitial pneumonia with the presence of edema, fibrin deposits, hemorrhages, and cell debris in alveoli. In the heart, a chronic fibrous pericarditis was described. In pig F5, although ASFV antigen was not detected by IHC in lung samples, histopathologic evaluation revealed the presence of chronic interstitial pneumonia along with chronic fibrous pleuritis. No histopathological changes were observed in the heart of pig F5. Necropsies of control nonimmune pigs (group G) showed gross lesions typical of acute ASF, including lymph nodes increased in size, edematous, and with hemorrhages, some of them resembling blood clots, hyperemic splenomegaly, erythematous tonsils, hydropericardium, and failure of the lungs to collapse (Fig. 7C).

### Levels of virus in blood.

**FIG 8:**
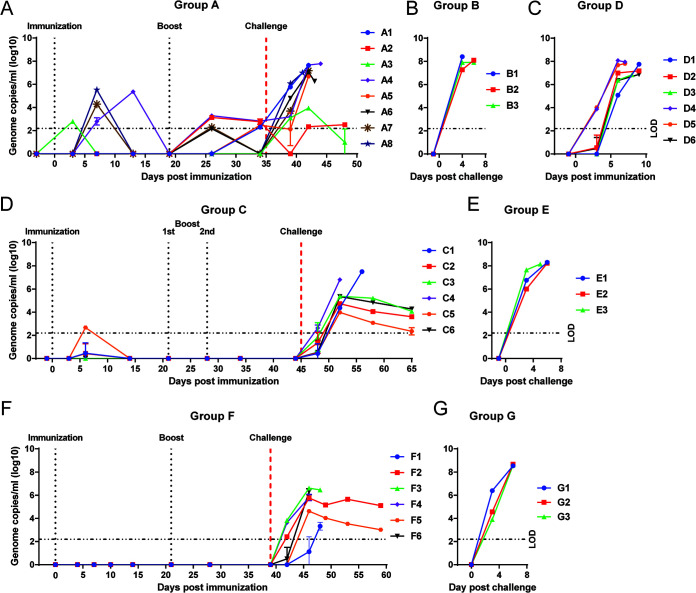
Viremia of pigs from experiments 1, 2, and 3. (A, C, D, F) Viral genome copies/mL in blood collected in EDTA were determined by quantitative PCR (qPCR) for pigs immunized with ΔMGF (group A, panel A), ΔMGF(B) (group D, panel C), ΔMGF(A) (group C, panel D), and Δ1R12L (group F, panel F). (B, E, G) Viremia for the nonimmune, challenge control groups for each experiment were recorded following challenge with Georgia 2007/1 isolate. The dashed and dotted line on the *y* axis represents the cutoff where accurate measurement viral genome copies/mL blood can be obtained. Any value below this indicates the presence of ASFV genome, but the exact titers cannot be determined.

Virus genome copies per mL of whole blood were determined for all pigs from the three different experiments at different days postimmunization. In pigs immunized with ΔMGF (group A), virus was detected in all eight pigs intermittently after immunization and before challenge. Pigs A4, A7, and A8 had at peak moderate levels of 10^4^ to 10^5^ virus genome copies/mL in blood at 7 or 13 dpi (Fig. 8A). Other pigs had low levels of virus genome of between 10^2^ and 10^3^ copies/mL. After challenge, all nonsurviving pigs had peak genome levels between 10^6.6^ and 10^7.7^ per mL (Fig. 8A). The surviving pig A3 had genome copies of 10^3^ to 10^4^ from 4 to 7 dpc, whereas A2 had very low levels of detectable genome (∼10^2^ genome copies/mL) postchallenge. As expected, the pigs in control group B had genome levels per mL rising above 10^8^ at termination (Fig. 8B).

Similar to group B controls, group D pigs immunized with 10^4^ HAD_50_ ΔMGF(B) and subsequently euthanized at a moderate severity endpoint between 7 and 9 dpi had high levels of virus genome in blood peaking at 10^6.9^ to 10^8.1^ copies/mL (Fig. 8C). This included pig D2, which had declining clinical scores (Fig. 4D). In contrast, viral genome was detected only in three group C pigs immunized with ΔMGF(A): one of these (C5) had viral genome slightly above 10^2^ genome copies/mL (Fig. 8D). Two of the pigs (C1 and C2) had genome levels below the cutoff value and thus could not be accurately measured. Beyond 6 dpi, viral genome was not detected before challenge in this group (Fig. 8D). After challenge, at 3 dpc, the control pigs in group E all had high levels of virus genome in blood (10^6^ to 10^7^ genome copies/mL) (Fig. 8E). When they were culled at 5 and 6 dpc, all three control pigs had approximately 10^8^ genome copies/mL. In contrast, all pigs from group C, had low levels of virus genome in blood at 3 dpc (Fig. 8D). Pig C4 had 10^6.8^ genome copies/mL at 7 dpc, at euthanasia. Pig C1 had 10^4.3^ genome copies/mL at 7 dpc, and this increased to about 10^7.5^ viral genome copies/mL at euthanasia at 11 dpc. All four surviving pigs had decreasing levels of viral genome from 7 dpc and by 20 dpc, ranging from approximately 10^2.4^ to 10^4.3^ genome copies/mL in all four pigs (Fig. 8D).

No virus genome was detected in blood from group F pigs after immunization with Δ1R12L and before challenge (Fig. 8F). At 3 dpc, three pigs in group F had low levels of virus in the blood (10^2^ to 10^3^ genome copies/mL). At 7 dpc, presence of ASFV viral genome was detected at levels below the cutoff value for accurate measurement in pig F1 (∼88 genome copies/mL), while the other pigs had moderate to high levels of virus genome (F2, F4, and F5: 10^4^ to 10^5^ genome copies/mL; F3 and F6: 10^6^ genome copies/mL) (Fig. 8F). At euthanasia on 9 dpc, pigs F1 and F3 had 10^3^ and 10^6^ viral genome copies/mL of blood, respectively. Pig F2, which survived to the end of the experiment, had a consistent level of virus until the end of experiment (10^5^ genome copies/mL), while pig F5 had decreasing amounts of viral genome detected from 49 dpi (10^4^) to 59 dpi (10^3^). The control pigs (group G) for this experiment, at the point of termination, had high level of virus in the blood (10^8^ ASFV genome copies/mL) (Fig. 8G). Our experience from previous experiments (21, 22) is that virus genome copy numbers in whole blood measured by quantitative PCR (qPCR) give very similar values to those obtained by virus titration in primary PBMs. The only exception found was in situations in which virus persisted for lengthy periods (for example greater than 15 or 20 days) without replication in the red blood cell fraction. In this case genome copy numbers may be higher than infectious virus levels. However, in the current experiments, longer-term persistence of virus was not observed.

### Antibody response against ASFV in pigs immunized with gene-deleted ASFV.

Pig antibody responses to ASFV p72/B646L protein for groups A, C, and F were measured using a blocking ELISA in sera extracted at different days postimmunization. In this assay, only samples showing blocking greater than 50% were considered positive. The pigs immunized with ΔMGF (group A) had no detectable antibodies above the cutoff threshold after immunization (Fig. 5A). After the boost, at 26 dpi, the two pigs that survived the challenge, pigs A2 and A3, had detectable antibody responses. Before the challenge, pig A4 also had a detectable antibody response, while the rest of the immunized animals remained doubtful or below the threshold. By 6 or 7 dpc, all pigs in group A were positive for p72 antibodies (Fig. 5A), including those that reached the moderate end points. The pigs in group C, immunized with ΔMGF(A), also mounted a delayed antibody response. No antibodies above the cutoff threshold were detected after the first immunization, and only three pigs had detectable antibodies a week after the first boost. After the second boost with a higher dose of the same virus, five pigs had positive antibody responses above the cutoff, and these levels continued to increase and plateaued after the challenge (Fig. 5B). However, pig C4 failed to mount an antibody response above the threshold until 7 dpc, the day it was euthanized. The antibody response in group F, immunized with Δ1R12L, developed more slowly than group C. Only three pigs had positive antibody responses after boost (Fig. 5C). Three days after challenge, pig F6 had a response above the threshold, while the two surviving pigs, F2 and F5, had an antibody response above the threshold on 7 dpc. The antibody blocking percentage for the pigs that showed an antibody response before challenge remained low even at 3 dpc, but this response was significantly boosted at 7 dpc.

### ASFV-specific IFN-γ-producing cellular response in pigs immunized with gene-deleted ASFV.

The presence of ASFV-specific IFN-γ-producing cells has previously been correlated with the level of protection induced by attenuated ASFV isolates (23). Here, PBMCs from group A, C, and F pigs, that were vaccinated with ΔMGF, ΔMGF(A), and ΔMR12L, respectively, were evaluated for the number of IFN-γ-producing cells induced by ASFV at three different time points: preimmunization, preboost, and prechallenge.

After immunization with ΔMGF and before the boost at 19 dpi, only three pigs from group A (pigs A2, A3, and A5) had increased numbers of IFN-γ-producing cells induced by stimulation of PBMCs with ASFV Georgia 2007/1 strain compared to the numbers induced before immunization (Fig. 6A). At 34 dpi, before challenge with virulent Georgia 2007/1, the number of IFN-γ-producing cells increased in all eight pigs compared to before immunization and boost (Fig. 6A). The pigs that survived the challenge, pigs A2 and A3, were among those in which the highest number of IFN-γ-producing cells were detected. Nevertheless, pigs A4 and A5, which did not survive the challenge, had similar levels of IFN-γ-producing cells induced by ASFV stimulation (Fig. 6A).

After immunization with ΔMGF(A), at 21 dpi, before the first boost immunization, low levels of IFN-γ-producing cells were induced in pigs C1, C2, and C3, and responses were barely detectable in the other three pigs (C4, C5, and C6). Based on these results, this group was boosted on 28 dpi with a higher dose. The number of IFN-γ-producing cells measured before challenge showed a significant increase in the ASFV-specific response in all six pigs (*P* = 0.002) (Fig. 6B). Nevertheless, the frequency of IFN-γ-producing cells did not correlate to the degree of protection. Pig C4 (euthanized at 7 dpc) had approximately 428 spot-forming cells (SFCs)/million PBMCs, while pig C1 (euthanized at 11 dpc) had the highest response among the six pigs in group C (∼631 SFCs/million PBMC).

Similar to ΔMGF(A)-immunized pigs, pigs immunized with Δ1R12L (group F) also had a low ASFV-specific IFN-γ response before boost; only two pigs, F1 and F6, had a significant increase in the number of IFN-γ-producing cells detected compared to their preimmune state (Fig. 6C). The booster dose was therefore given at a higher dose on 21 dpi. Before challenge, PBMCs from all six pigs had a significant increase in the numbers of IFN-γ-producing cells following stimulation with Georgia 2007/1 compared to levels detected before immunization (*P* < 0.02) (Fig. 6C). The number of IFN-γ-producing cells detected following stimulation of PBMCs from pigs F5 and F6 did not change significantly in levels between preboost and prechallenge. Pigs F2 and F5, which survived the challenge virus, had the lowest numbers of IFN-γ-producing cells at 39 dpi (Fig. 6C).

Comparing of the numbers of ASFV-specific IFN-γ-producing cells induced by these three gene-deleted viruses, ΔMGF, ΔMGF(A), and Δ1R12L, showed no significant differences after just one immunization at either 19 or 21 dpi (Fig. 6D). Following the booster immunization with the same virus, an overall increase was noted in the numbers of IFN-γ-producing cells stimulated by ASFV, and PBMCs from pigs in group C responded to a significantly higher level compared to those in group F (*P* < 0.05) (Fig. 6D) line.

## DISCUSSION

ASFV codes for various numbers of genes belonging to five different MGFs. Deletion of multiple copies of genes belonging to multigene families MGF360 and MGF505 has been correlated with a decrease in virus virulence, induction of variable levels of protection against challenge, and an increased type I IFN response in macrophages infected in culture (6, 8). However, it remained unknown whether some MGF360 or MGF505 genes had a greater impact on the virus attenuation and induction of protection than others. Here, we investigated the impact of deleting different combinations of MGF360 and MGF505 genes from the virulent genotype II ASFV Georgia 2007/1 isolate. This isolate was obtained from the first introduction of genotype II to Georgia in 2007. Many genotype II isolates circulating in Europe and Asia are highly virulent, although some of reduced virulence have been reported from Russia, the Baltic States, Poland, and China (24–29). The results we obtained are summarized in Table 1.

In previous studies, we demonstrated that deletion of MGF360-10L, -11L, -12L, -13L, and -14L and MGF505-1R, -2R, and -3R and the interruption of MGF360-9L and MGF 505-4R from the genotype I, Benin 97/1 isolate did not reduce virus replication in macrophages. This deletion attenuated the Benin 97/1 isolate, although moderate clinical signs and low-to-moderate viremia were detected after immunization. Levels of protection between 66 and 100% were obtained when pigs were challenged with the parental virulent isolate, depending on the route and dose of the BeninΔMGF virus administered (6, 20). The results obtained in the present study show that virus, ΔMGF, with a similar deletion from the genotype II Georgia 2007/1 isolate had reduced replication in macrophages and was attenuated in pigs. No clinical signs were observed postimmunization, although viremia up to 10^5^ genome copies/mL were occasionally observed in some pigs following immunization. A lower level of protection (25%) was observed following challenge with virulent Georgia 2007/1 virus compared to that observed with the BeninΔMGF deletion. Deletion of genes MGF360-9L, -10L, -11L, -12L, -13L, and -14L and MGF505-1R and -2R from the virulent Pr4 strain also reduced growth of the virus in macrophages (30). This provides further evidence to suggest that the impact of deleting these genes on growth in macrophages may be strain specific. There may be differences in the functions of individual MGF360 and MGF505 proteins between the strains, although variation in function of other encoded proteins is possible. In studies from other authors (7), deletion of six genes belonging to MGF360 or MGF505: MGF505-1R, MGF360-12L, MGF360-13L, MGF360-14L, MGF505-2R, and MGF505-3R was sufficient to attenuate the virulent ASFV genotype II Georgia isolate and induce protection against challenge with parental virulent virus. However, low to moderate levels of virus genome were detected postimmunization (maximum 10^5^ to 10^6^ HAD_50_/mL). In this study, the authors did not determine whether deletion of any of these genes was particularly important for virus attenuation or if all contributed (7).

In our study, we investigated whether deleting some of the MGF360 or MGF505 genes deleted from GeorgiaΔMGF had a greater impact on virus replication in macrophages or attenuation in pigs, by constructing and testing five mutants with deletions in members of MGF360 and MGF505. These mutants were constructed from ASFV Georgia 2007/1 that had the K145R gene deleted. The K145R gene has been shown to code for an abundant late cytoplasmic protein (18, 31) and is a potential negative serological marker for diagnostics to differentiate infected from vaccinated animals (19). We recently showed that deletion of K145R had only a mild effect on attenuation of the Georgia 2007/1 strain because a delay but not a reduction in clinical signs was observed postimmunization (17).

In the current study, we showed that deletion of different subsets of MGF360 and MGF505 gene(s) had various impacts on virus growth rates in PBMs. This was correlated with the deletion of the MGF360-12L gene as observed in cell infections with ΔMGF(A), Δ1R12L, and Δ12L. The ΔMGF(B) virus, which has deletions of MGF360-13L and -14L and MGF505-2R and -3R, initially replicated slower in macrophages; however, by 3 days postinfection, no significant differences in virus titers recovered were detected compared to wild-type Georgia 2007/1 ASFV. Although these viruses also had the K145R gene deleted, the reduced replication correlated with deletion of MGF360-12L, suggesting that deletion of this gene had the major impact. In contrast, others have shown that other genotype II viruses with six copies of MGF360 and 505 deleted, including MGF360-12L, MGF360-13L and -14L, and MGF505-1R, -2R, and -3R, had growth kinetics similar to those of the genotype II wild-type virus (7, 32). The difference between our results and others may be related to the additional deletion of the K145R gene in the current study, although we did not observe a growth defect for the Δ1R virus. Differences in the primary cell culture conditions might also provide an explanation for differences between our and previous studies.

The mechanism of action of the proteins encoded by MGF360 and MGF505 genes is poorly understood. A large deletion of MGF360 and MGF505 genes from the Pr4 or Benin 97/1 isolates was shown to result in an increase in type I IFN and IFN-stimulated gene (ISG) expression, as well as increased sensitivity of virus replication to pretreatment with type I IFN (6, 8, 33). Here, we showed that higher levels of IFN-α and the interferon-stimulated chemokine, CXCL10, were secreted into the medium of PBMs infected with the Δ12L deletion mutant and a lower level into media from the double deletion mutant Δ1R12L. A further reduction in levels of IFN-α and CXCL10 were detected in media from PBMs infected with other deletion mutants. Thus, deleting genes in addition to MGF360-12L appeared to result in reduced IFN-α and CXCL10 secretion compared to the single MGF360-12L deletion. Although we do not have an explanation for these results, one possibility is competition between different MGF360 and 505 proteins of various inhibitory activity, for factors involved in the relevant host signaling pathways. We tested induction of CXCL10 as an example of ISG and further predicted that a broad range of ISGs would be induced in cells infected with deletion mutants of selected MGF360 and 505 genes. This could contribute to activation of host restriction factors to limit replication in cells and of innate and adaptive immune responses in immunized pigs. One possibility is that the reduced replication that we observed in PBMs infected with deletion mutants, including ΔMGF, ΔMGF(A), Δ1R12L, and Δ12L, may be due to an IFN-stimulated activation of host restriction factors to limit virus replication. This hypothesis requires further investigation.

The impact of the gene deletions on replication in cells was well correlated with reduction in virulence. Thus, the ΔMGF(B) virus was not attenuated when tested in pigs since, despite a slight delay in the appearance of clinical signs, all pigs had high levels of viremia, and all reached a moderate severity humane endpoint between 7 and 9 dpi. In another study deletion of MGF360-13L and MGF360-14L did not attenuate a virulent genotype II Georgia 2007 virus (34). Here, we show that the additional deletion of MGF505-2R and 3R does not attenuate the virus significantly. In contrast, ΔMGF(A) virus did not induce any clinical signs in pigs after immunization, and only one pig had a low virus genome copy number detected early postimmunization. Since genes MGF360-13L and MGF360-14L were deleted from both ΔMGF(A) and ΔMGF(B), we concluded that deletion of the MGF360-12L and MGF505-1R genes, in combination with K145R, should be sufficient to attenuate the virus. This was confirmed since pigs immunized with Δ1R12L did not develop any clinical signs, and no viral genome was detected before the challenge.

The ΔMGF virus has additional deletions/interruptions of MGF360-9L, -10L, and -11L and MGF505-2R, -3R, and -4R genes compared to the ΔMGF(A) deletion. Deletion of these additional genes may explain the reduced replication of ΔMGF compared to the ΔMGF(A), Δ1R12L, and Δ12L viruses. The reduction in virus replication in macrophages correlated with increased attenuation in pigs. While no clinical signs were observed following immunization of pigs with either ΔMGF, ΔMGF(A), or Δ1R12L, the levels of protection, defined as causing clinical signs that did not exceed the moderate severity humane endpoint used in the studies, varied between different immunizing viruses. Thus, ΔMGF and Δ1R12L viruses induced 25 and 33.3% protection, respectively, whereas ΔMGF(A) induced 66.7% protection against virulent challenge virus. ΔΔMGF and Δ1R12L replicated with very similar kinetics and to the same titers in macrophages; thus, the level of replication in macrophages alone did not explain the differences in protection. Our results indicate that the deletion of MGF360-13L and -14L, in addition to MGF360-12L and MGF505-1R, the combination deleted from ΔMGF(A), enhances the induction of a protective immune response. Although the deletion of the MGF360-12L gene appears to be critical for virus attenuation, further genes may need to be deleted to enhance the immune response.

In support of this hypothesis, our results showed that pigs immunized with ΔMGF(A) induced both stronger ASFV-specific antibody responses and cellular responses as measured by IFN-γ ELIspot assays compared to those immunized with Δ1R12L. It was shown previously that depletion of CD8^+^ cells abrogated the protection induced by the attenuated strain OURT88/3 (35), indicating that a CD8^+^ cellular response is required for protection. Passive transfer of antisera from protected to naive pigs conferred protection against lethal challenge (36). The mechanisms by which antibodies confer protection is unclear since neutralizing antibodies are inconsistently detected (for review see [37, 38]). In our experiments, better protection was observed when higher levels of both ASFV-specific antibodies and IFN-γ-producing cells were induced, consistent with both arms of the immune response playing a role in protection.

The present results provide the first indication that deletion of just one member, MGF360-12L, of MGF360 or 505 gene families, in combination with K145R, can have a dramatic effect on reducing virus replication in cells and that deletion of MGF360-12L and MGF505-1R, in combination with K145R, results in strong virus attenuation in pigs. ΔMGF(A) and Δ1R12L have the K145R gene deleted in addition to the MGF genes. However, the ΔMGF(B) virus also has K145R gene deleted and showed only slight attenuation observed as a delay in observation of clinical signs. Thus, the K145R gene deletion is likely to have only a small effect on attenuation of viruses.

The MGF360-12L protein sequence is generally very well conserved within and between genotypes. The Georgia 2007/1 and Benin 97/1 MGF360-12L protein sequences are 93% identical and 97% conserved, although there may be some differences in function. It would be interesting in the future to immunize pigs with the Δ12L virus, since our results indicate this virus should also be attenuated but possibly may not induce a strong protective response. Further research is needed to better understand the roles of individual MGF360 and MGF505 proteins during virus replication in cells and in infected pigs. Our results show that the combination of MGF genes deleted from the ΔMGF(A) virus, MGF360-12L, -13L, and -14L and MGF505-1R is a promising combination for further development of safe and effective vaccine candidates. Although the ΔMGF(A) virus appears to have an excellent safety profile, ideally efficacy should be improved. This may be achieved by administration of higher doses. Alternatively, deleting additional genes to enhance the immune response without further virus attenuation could be effective. A more thorough understanding of the role during infection of MGF360 and MGF505 and other immune evasion genes is required to tailor this response. The additional deletion of the K145R gene provides a potential negative serology marker to distinguish vaccinated from infected pigs, a further important step for the eventual field use of vaccines. Advancing our knowledge of the MGF gene family proteins will provide a crucial step forward to understanding the various pathogenesis and immune responses induced by different ASFV isolates and enable this knowledge to be applied to rational vaccine design.

## MATERIAL AND METHODS

### Viruses and cells.

A genotype II ASFV virulent isolate, Georgia 2007/1, described previously (39) was used to generate recombinant viruses. The deletion of K145R from GeorgiaΔDP148R was described previously; here, similarly K145R was deleted from the Georgia 2007/1 singly from positions 64734 to 65086 leaving 85 bp at the 3′ terminal where the promoter for the adjacent K421R gene may lie (17). ASFV was propagated in porcine bone marrow cells (PBMs) cultured in Earle’s balanced salt solution (EBSS) supplemented with 10% porcine serum and 1% penicillin-streptomycin (pen-strep) or in purified PBMs maintained in RPMI supplemented with 10% fetal bovine serum (FBS), 1% pen-strep, and 100 ng/mL porcine CSF1 (Roslin Tech). PBMs were obtained from the leg bones of 4- to 5-week old outbred Large White Landrace cross pigs. These PBMs were either used as they were or further purified by density gradient centrifugation using Histopaque-1083, at 1.083 g/mL to extract the mononuclear cell fraction and remove erythrocytes. The purified PBMs were used mainly in the process of producing the recombinant viruses. Porcine alveolar macrophages (PAM) were obtained by pig lung lavages and were cultured in RPMI supplemented with 10% porcine serum and 1% pen-strep. WSL-R, a wild boar cell line, was grown in ZB28 medium (50% Ham’s F12 nutrient mix medium, 50% Iscove’s modified Dulbecco’s medium [IMDM]) supplemented with 10% FBS, 1% pen-strep, and 1% l-glutamine. PAM or WSL-R were used only during the initial homologous recombination step while producing the recombinant ASFV (40). Virus titrations were carried out in quadruplicate on PBMs. Titers were calculated using the Spearman and Kärber algorithm (41) and are expressed as HAD_50_/mL (42).

### Recombinant viruses.

Genetic modification of ASFV was ethically reviewed and carried out under license from the United Kingdome Health and Safety Executive. Six recombinant gene-deleted ASFV were produced by homologous recombination between (i) transfer plasmids containing left and right flanking regions of the gene(s) to be deleted and reporter genes under the control of ASFV promoters and (ii) a parental virus (Fig. 1).

Genes MGF360-10L, MGF360-11L, MGF505-1R, MGF360-12L, MGF360-13L, MGF360-14L, MGF505-2R, and MGF505-3R were deleted, while MGF360-9L and MGF505-4R were interrupted from the virulent Georgia 2007/1 isolate (Fig. 1A) to produce GeorgiaΔMGF (addressed as ΔMGF here) (Fig. 1B). To construct the transfer plasmid, pΔMGF-VP72GUS, flanking regions of MGF360-9L (left) and MGF505-4R (right) were amplified by PCR and cloned upstream or downstream of the β-GUS gene cassette under the control of the ASFV VP72 promoter (43). PAMs were infected with Georgia 2007/1 and subsequently transfected with pΔMGF-VP72GUS. Five days after infection-transfection, cells expressing the reporter GUS were detected via addition of X-GLUC, and these GUS-positive wells were purified by multiple rounds of limiting dilution in PBMs.

For the remaining recombinant viruses, the transfer plasmids were synthesized to harbor mNeonGreen (44), a fluorescent protein reporter gene under the control of ASFV VP30 promoter and 500 to 685 bp of the left and right flanking arms of the gene(s) to be deleted (GenScript, USA). WSL-R cells were infected with GeorgiaΔK145R, an ASFV isolate with K145R deleted, and containing a TagRFP-T reporter protein (17, 45), and then transfected with the synthesized transfer plasmids. Single cells expressing both red (ΔK145R) and green (ΔMGFs) fluorescent proteins were isolated via fluorescence-activated cell sorting (FACS) into purified PBMs (46). All five recombinant viruses were then purified via two rounds of single cell sorting combined with two rounds of limiting dilutions (46). These recombinant viruses have different MGF genes deleted and include GeorgiaΔK145RΔMGF(A) (addressed as ΔMGF(A) here), which has MGF360-12L, MGF505-1R, MGF360-13L, and MGF360-14L deleted (Fig. 1C). GeorgiaΔK145RΔMGF(B) (addressed as ΔMGF(B) here) has the MGF360-13L and -14L and MGF505-2R and -3R genes deleted (Fig. 1D). Three other recombinant viruses were subsequently produced to have MGF505-1R and/or MGF360-12L deleted, in conjunction (Fig. 1E) and singly (here addressed as Δ1R12L, Δ1R, and Δ12L, respectively) (Fig. 1F and 1G).

Viral DNA was extracted after the last round of single cell sorting and after each limiting dilution step using MagVet universal isolation kit (Thermo Fisher Scientific), and the high-throughput KingFisher flex extraction system (Thermo Fisher Scientific). PCR amplifications were performed to confirm the deletion of target gene(s) and the absence of parental virus. The PCRs also confirmed that insertions were at the correct genome position by amplification from a primer in a region outside the flanking region of the transfer plasmid to within the reporter gene. Finally, the whole recombination site was subjected to Sanger sequencing to confirm the expected deletion and correct insertion of reporter gene cassette.

### Multistep growth curve.

To measure growth over multiple rounds of infection, ASFV wild-type isolate, Georgia 2007/1, and recombinant viruses were added to purified PBMs at a multiplicity of infection (MOI) of 0.01 in triplicate in 24-well plates. After an hour of incubation at 37°C, the inoculum was removed, and the infected cells were washed once gently with Dulbecco’s phosphate-buffered saline (PBS), before adding fresh complete medium. The cells and supernatants were harvested every 24 h for 5 days and freeze-thawed thrice. The experiment was carried out in purified PBMs from two different pigs, and all samples were titrated as described above. Repeated measure two-way analysis of variance (ANOVA), where each row represented different days of infection, was used to evaluate the differences between the titers of different viruses over time. Dunnett’s multiple-comparison test was performed to evaluate the mean differences between the six recombinant viruses against the parental wild-type virus.

### Virus purification.

PBMs were infected with recombinant and wild-type viruses and incubated for 4 days at 37°C, with 5% CO_2_. Supernatants were collected and clarified at 1,000 × *g* for 10 m at 4°C. To separate virus particles from smaller molecules (i.e., cytokines), viruses were purified via filtration using the 1,000,000 molecular weight cutoff (MWCO) PES Vivaspin filter spin column (Vivaspin 20, Sartorius). The filters were first sterilized with 70% ethanol and washed twice with PBS. Virus supernatants were then added and centrifuged at 3,500 × *g* for 1 h. The concentrated virus retained in the upper chamber was then transferred into fresh tubes. The filters were rinsed with 2 mL complete medium, and this was combined with the virus collected from the first centrifugation. Purified viruses were titrated as described above.

### Quantification of IFN-α and CXCL10 levels in virus-infected supernatant.

Purified PBMs from two outbred pigs were seeded at 1 × 10^6^ cells/mL and infected with purified recombinant and wild-type viruses at a MOI of 0.5 in duplicate. After 1-h incubation at 37°C, the inoculum was removed, and fresh medium was added; infected cells were further incubated for 16 h at 37°C. The supernatants were collected and centrifuged at 300 × *g* for 5 min to remove cells. The levels of IFN-α in these supernatants were then evaluated using an in-house ELISA (47). First, Maxisorp plates (Nunc) were coated with anti-pig IFN-α antibody (clone K9) at 0.5 µg/mL in 0.05 M coating buffer overnight at room temperature, and subsequently the plates were washed with 0.05% Tween 20 in PBS and blocked with 1% bovine serum albumin (BSA) in PBS. Standards (recombinant porcine IFN-α, PBL Assay Science) and samples were then added in duplicate and were then incubated at room temperature for 2 h. After washing, biotinylated anti-pig IFN-α antibody (clone F17) was diluted 1:5,000 in blocking buffer and added to the plates. After a further 2 h of incubation at room temperature, the plates were washed, incubated with streptavidin-horseradish peroxidase (HRP), and finally developed with 3,3′,5,5′-tetramethylbenzidine (TMB) substrate (R&D Systems, DY999). After stopping the reaction with 2 N H_2_SO_4_, the absorbances were read at 450 nm.

The concentration of CXCL10 in the supernatant was quantified using a swine CXCL10 Do-It-Yourself ELISA (Kingfisher Biotech). Briefly, Maxisorp plates were coated with capture antibody (anti-swine CXCL10 [IP-10] polyclonal antibody, PB0119S-100) at 2.5 μg/mL in PBS overnight. The plates were washed thrice with PBS-T (0.05% Tween 20 in PBS) and blocked with 4% BSA for 1 h at room temperature. Samples and standards were then added, and the plates were incubated at room temperature for 1 h. After four washes with PBS-T, detection antibody (biotinylated anti-swine CXCL10 [IP-10] polyclonal antibody, PBB1138S-050), diluted in blocking buffer at 0.05 μg/mL, was added to plates and incubated for another hour at room temperature. Following four rounds of washes, streptavidin-HRP was added and finally developed with TMB substrate. The reaction was stopped with 2 N H_2_SO_4_, and plate absorbance was read at 450 nm.

### Pig immunization and challenge experiments.

**FIG 9:**
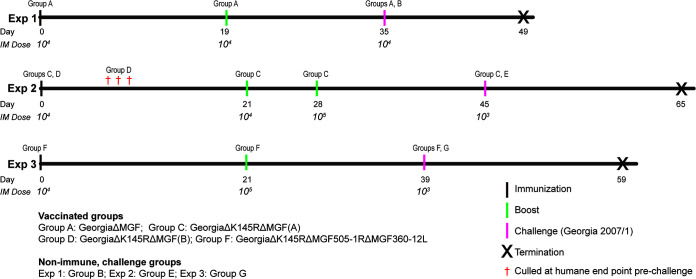
Schedules for the pig immunization and challenge experiments. The schedules and doses of viruses used for immunization, boost, and challenge in the three pig immunization experiments are shown. The days of immunization (black bars), boost(s) (green bars), and challenge with virulent ASFV Georgia 2007/1 (pink bars) and day of termination of the experiment (**×**) are shown. The doses (HAD_50_) of gene-deleted and challenge viruses are shown under the days the doses were administered. All were given to pigs in 1 mL intramuscularly. In experiment 1, the pigs were immunized and boosted with ΔMGF (group A). In the second experiment, the pigs were immunized with ΔMGF(A) (group C) or ΔMGF(B) (group D). In experiment 3, the pigs were immunized with Δ1R12L (group F). In each experiment groups of nonimmune control pigs were challenged at the same time as the immunized groups (groups B, E and G). The red daggers (†) denote pigs that were culled upon immunization.

All animal experiments were conducted at the SAPO4 high containment animal housing at the Pirbright Institute (Woking, UK). Experiments were carried out in accordance with the regulated procedures from the Animals (Scientific Procedures) Act UK 1986 and conducted under Home Office License 7088520. All pigs were female Large White Landrace cross, weighing between 15 and 23 kg. Titers of the virus inoculum were confirmed by backtitrations. Clinical signs, including temperatures, lethargy, anorexia, respiratory distress, vomiting, signs of skin hemorrhage, or bloody diarrhea, were recorded daily throughout the experiment and scored as described previously (48). The pigs were terminated if they reached a moderate severity humane endpoint as defined in the Home Office License. This endpoint was reached when temperatures above 40.6°C were reached for 4 consecutive days or for 3 days if other signs, including anorexia or morbidity, were observed. Scoring of gross lesions typical of ASFV infection was performed at necropsy (49, 50). Three experiments were carried out as outlined below. All immunizations and challenges were carried out by the intramuscular route. Details of the experimental design are described in the Results section and shown in Fig. 9.

In experiment 1, the pigs were immunized and boosted with 10^4^ HAD_50_ ΔMGF, followed by challenge with virulent Georgia 2007/1 (group A). A control group of nonimmune pigs (group B) was included at challenge. In experiment 2, immunizations of pigs with ΔMGF(A) (group C) and ΔMGF(B) (group D) were compared, and challenge of group C with virulent Georgia 2007/1 was carried out in parallel with a control group E of nonimmune pigs. In the final experiment, the pigs were immunized with Δ1R12L (group F) and challenged with virulent Georgia 2007/1 virus in parallel with a group of nonimmune control pigs (group G).

### Measurement of viral genome in blood.

Whole blood samples were collected in EDTA at different days during the experiments. Viral nucleic acid was extracted using MagVet Universal isolation kit and the automated KingFisher Flex extraction system (Thermo Fisher Scientific). Samples from different days were extracted in duplicate, and the extracted DNA was then subjected to qPCR as described previously (51). The results are presented as genome copies/mL blood. A cutoff for accurate detection was based on the serial 10-fold dilutions of a control plasmid, where the lowest value is 1 copy. This translates to a cutoff accurate measurement of 1.59 × 10^2^ ASFV genome copies/mL. Below this, the exact genome copy cannot be determined. We defined levels of virus genome less than 10^4^ genome copies/mL as low, 10^4^ to 10^6^ genome copies/mL as moderate, and more than 10^6^ as high.

### ELISA to detect ASFV-specific antibodies.

The antibody responses of the immunized pigs were measured using serum extracted on different days postimmunization using a blocking ELISA against ASFV VP72 protein (INgezim PPA COMPAC, Ingenasa) as described by the manufacturer. The optical density (OD) was read at 450 nm on a microplate reader BioTek with Gen5 software. The percentage of blocking was calculated using the following equation, $\frac{\left(\mathrm{negative}\;\mathrm{control}\;\mathrm{OD}-\mathrm{sample}\;\mathrm{OD}\right)}{\mathrm{negative}\;\mathrm{control}\;\mathrm{OD}-\mathrm{positive}\;\mathrm{control}\;\mathrm{OD}}\times 100\%$. Samples above 50% blocking were considered positive, while anything below 40% was considered negative. Samples with blocking between 40 and 50% were considered doubtful.

### IFN-γ ELISpot assay.

Whole blood was collected in EDTA before immunization, boost, and challenge, and the pigs’ peripheral blood mononuclear cells (PBMCs) were isolated using density gradient centrifugation with Histopaque-1083 (Sigma). MultiScreen-IP filter plates with 0.45-μm pore size (MAIPS4510, Millipore) were coated overnight at 4°C with 4 μg/mL capture anti-IFN-γ (P2F6, Thermo Fisher) in 0.05 M carbonate-bicarbonate coating buffer. Washed plates were then seeded with freshly isolated PBMC at two concentrations, 8 and 4 × 10^5^ cells/well, in RPMI 1640, GlutaMAX supplemented with 10% FBS, 1% of pen-strep (10,000 U/mL), and 50 μM β-mercaptoethanol. PBMCs were stimulated with Georgia 2007/1 ASFV at 10^6^ HAD_50_/mL, with an equivalent amount of mock-infected inoculum as a negative control and phytohemagglutinin (PHA) at 20 μg/mL as a positive control. The plates were then incubated at 37°C, for 16 to 18h, after which the cells were lysed in water for 5 min. Next, 1 μg/mL biotinylated anti-IFN-γ monoclonal antibody (P2C11, Thermo Fisher) was added, and the plates were incubated at room temperature for 2 h and then washed with PBS. Diluted streptavidin alkaline phosphatase conjugate (Invitrogen) was added, and the plates were further incubated for 1 h at room temperature. Lastly, alkaline phosphatase substrate (Bio-Rad) was added to develop the spots. SFCs were counted on an ELISpot reader (ImmunoSpot, CTL). Repeated measure two-way ANOVA, with either Tukey’s or Sidak’s multiple-comparison test, where each row represented days postimmunization, was used to evaluate the mean differences in the number of IFN-γ-producing cells over time within each group and between groups at prebooster and prechallenge, using GraphPad Prism version 7.00 for Windows (GraphPad Software, La Jolla, CA).
